# Ultrasound‐Assisted for Super‐Rapid and High‐Efficient Adsorption and Desorption

**DOI:** 10.1002/advs.202504905

**Published:** 2025-05-28

**Authors:** Yijun Han, Quanjie Lv, Linxuan Zhang, Yuruo Zhang, Xinyue Yu, Yongjie Wu, Jing Chu, Gengxin Zhang, Kang Sun, Ke Tao

**Affiliations:** ^1^ State Key Lab of Metal Matrix Composites School of Materials Science and Engineering Shanghai Jiao Tong University Shanghai 200240 P. R. China

**Keywords:** desorption, piezoelectric, PVDF, rapid adsorption, ultrasound

## Abstract

Adsorption–desorption technology is the basis of numerous environmental processes and industries in modern society. Undoubtedly, improving adsorption rates and renewability of sorbents can positively impact productivity. It is herein showed that poly(vinylidene fluoride‐co‐hexafluoropropylene) (PVDF‐HFP) presented remarkable adsorption kinetics and regeneration efficiency under ultrasound. The pseudo‐first‐order adsorption rate constant for Rhodamine B (RhB) is 2.7753 min^−1^, with an initial adsorption rate 7,000–23,000 times higher than commercial activated carbon. Meanwhile, the polymer achieves over 90% removal of the antibiotic levofloxacin (LEV) and the persistent pollutant perfluorooctanoic acid (PFOA) within 5 min. Impressively, complete desorption occurs within 2 min by simply changing the solvent, and the performance is retained across at least 20 adsorption–desorption cycles. It is proposed that, under ultrasound, the low interface tension of PVDF‐HFP may accelerate solute diffusion by disrupting the liquid film, while the ferroelectric PVDF‐HFP generates a built‐in electric field to enhance adsorption. This research suggested an alternative strategy for developing high‐performance adsorption–desorption techniques.

## Graphical abstract

Adsorption–desorption technologies face challenges due to slow adsorption and complex regeneration. This study shows that PVDF‐HFP offers exceptional adsorption kinetics and regeneration efficiency under ultrasound, adsorbs Rhodamine B 23,000 times faster than activated carbon, removing 90% of levofloxacin and PFOA in 5 min, and maintains performance for 20 cycles, due to its ferroelectric properties and low surface energy.

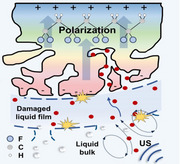

## 1 Introduction

Adsorption and desorption allow selective removal of contaminants, eliminating impurities and concentrating target substances. They are fundamental to numerous industrial and environmental processes, including water treatment, fine chemistry, pharmaceutical industries, mining engineering, etc.^[^ ^1^ ^]^ Adsorption also dictates the efficiency of catalysis, batteries, and energy storage.^[^ ^2^ ^]^ Meanwhile, desorption facilitates the regeneration of adsorbent materials to improve cost‐effectiveness and sustainability.^[^ ^3^, ^4^ ^]^ Classical sorbents and techniques relying on van der Waals and electronic interactions face unsatisfactory adsorption rates and desorption efficiency.^[^ ^5^ ^]^ Slow adsorption kinetics in water treatment systems result in long operational times and high costs, exemplified by the fact that several days are generally required for activated carbon to reach equilibrium in removing pollutants.^[^ ^5^, ^6^ ^]^ Energy penalties associated with adsorbent regeneration can account for up to 70% of the total operational costs in carbon capture technologies.^[^ ^7^ ^]^ Additionally, the structural degradation of sorbents, which typically occurs after five regeneration cycles, exacerbates material wastage and environmental burden.^[^ ^8^ ^]^ Thus, developing sorbent materials and techniques with enhanced kinetics, stability, and energy efficiency could essentially benefit the advancement of cleaner technologies, the conservation of resources, and the fostering of environmental sustainability.^[^ ^9^ ^]^

We noticed that the adsorption kinetics are dominantly limited by a traversing procedure in classical strategies, in which solute molecules must traverse a solvent film at the sorbent surface before being absorbed.^[^ ^10^ ^]^ We hypothesize that applying ultrasonics to specific sorbents might accelerate the traversal. Meanwhile, applying an electric field can enhance absorption efficiency and facilitate the control over adsorption and desorption.^[^ ^11^ ^]^ Fortunately, ultrasound can stimulate piezoelectric materials to form an intrinsic electric field. Poly(vinylidene fluoride ‐based piezoelectric polymers have attracted the attention of researchers as potential adsorbents,^[^ ^12^ ^]^ but their limited adsorption efficiency, the necessity of combination with other materials, and the unclear adsorption mechanism remain challenges.^[^ ^13^ ^]^ Thus, we propose that fluoro‐based piezoelectric polymers might be effective sorbents with the assistance of ultrasound. First, fluoro‐based polymers have low surface energy (10–40 mN m^−1^),^[^ ^14^ ^]^ which benefits the formation of interfacial cavitation under ultrasound to accelerate solute diffusion. Second, with the ultrasound‐polarized piezoelectric polymers, electrostatic forces would guide the migration of charged molecules toward the adsorbent surface. Therefore, we prepared poly(vinylidene fluoride‐co‐hexafluoropropylene) (PVDF‐HFP) membranes that harness the piezoelectric effect, showcasing exceptional efficiency and ultra‐fast adsorption–desorption performance of diverse molecules, including dyes, antibiotics, and perfluorooctanoic acid (PFOA). Fluorine content, pore structure, ferroelectricity, and surface energy were further adjusted to explore the mechanism.

## 2 Results and Discussion

### 2.1 Ultrafast Adsorption of Various Molecules by PVDF‐Based Materials

**Figure 1:**
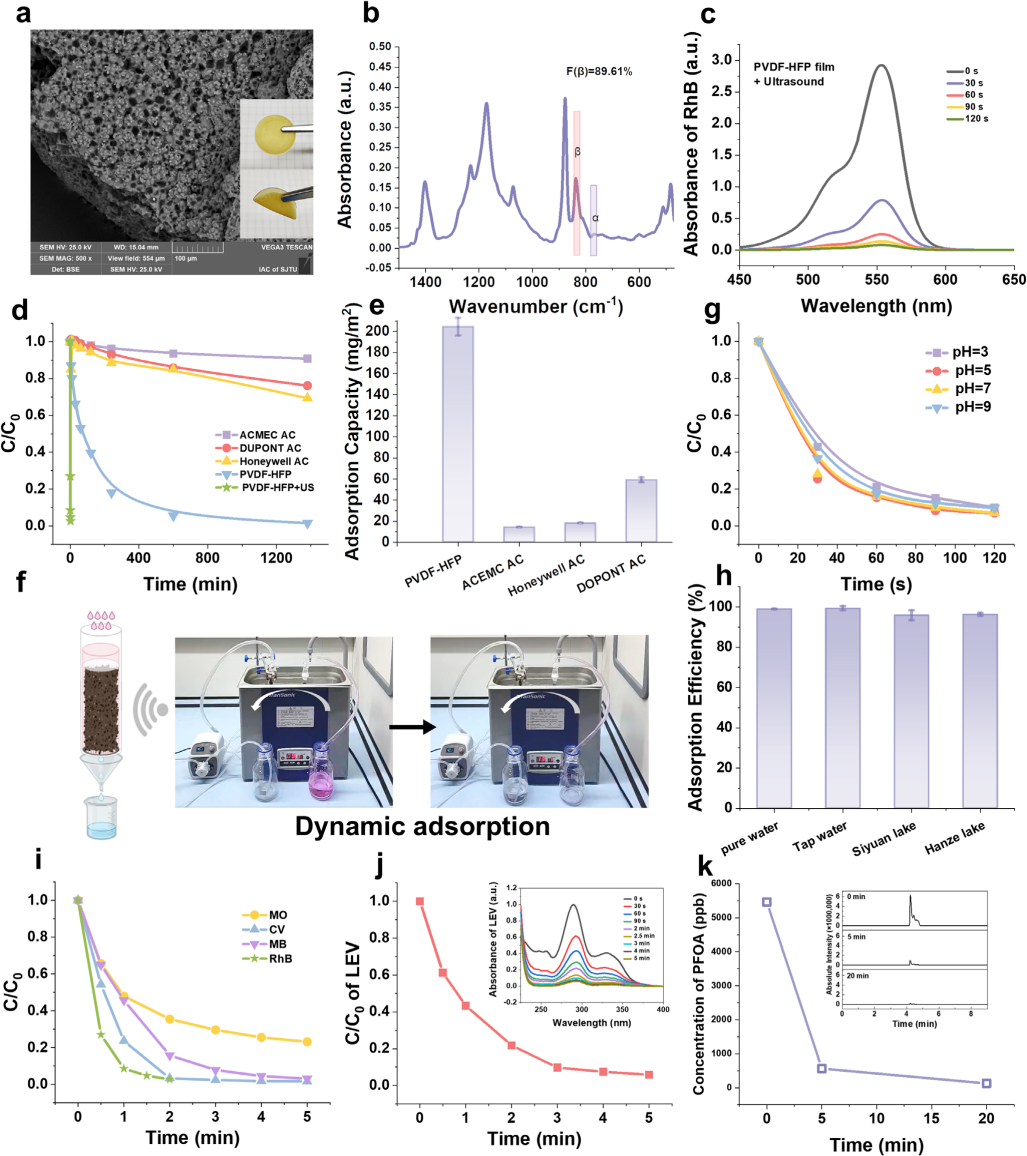
Preparation, morphology, and adsorption performance. a) Scanning electron microscope (SEM), photography (inset), and b) FTIR of the PVDF‐HFP material. c) Adsorption performance of RhB by PVDF‐HFP material under ultrasound. d) Time‐dependent adsorption of RhB (100 ppm) by PVDF‐HFP material and different brands of activated carbon (AC). e) Adsorption capacity per unit area of PVDF‐HFP material and activated carbon. f) Dynamic adsorption performance of PVDF‐HFP membranes. Adsorption performance of PVDF‐HFP membranes for RhB in g) various pH and h) aquatic environments. i) Adsorption efficiency of the PVDF‐HFP material for different pollutants within 5 min of ultrasonication. Adsorption performance of j) LEV and k) PFOA by PVDF‐HFP material under ultrasound.

PVDF‐HFP films were prepared via a phase inversion method^[^ ^15^ ^]^ followed by casting into molds (Figure S1, Supporting Information). The microstructure of PVDF‐HFP is shown in **Figure** **1**a, with a porosity of 73% determined by the mercury intrusion method. The film is flexible and easily bendable (Figure 1a inset), which allows it to withstand ultrasonic stimulation while maintaining structural integrity. Fourier‐transform infrared spectroscopy (FTIR) reveals that the ferroelectric β‐phase constitutes 89.61% (Figure 1b). Then, the adsorption kinetics of the membrane were evaluated by employing RhB as a model pollutant. The adsorption efficiency of a PVDF‐HFP material (diameter 22 mm, thickness 0.5 mm, 0.046 g) was assessed in a 100 ppm RhB solution (Figure 1c). Under static conditions, PVDF‐HFP achieved 82% adsorption in 4 h and 95% in 10 h. However, ultrasonic treatment significantly boosted the adsorption rate, reaching over 95% adsorption within just 90 s and nearly complete adsorption in 120 s (Figure 1d). By contrast, complete removal of the same dose of RhB in references typically required tens of minutes or even several hours^[^ ^16^ ^]^(Table S1, Supporting Information).

For comparison, the adsorption rates of commercial activated carbons (DUPONT, Honeywell, ACMEC) of equal weight were evaluated, with typical adsorption rate constants ranging from 10^−3^ to 10^−2^ min^−1[^ ^17^ ^]^ (Table S2, Supporting Information). Kinetic fitting showed that PVDF‐HFP materials under ultrasound followed the pseudo‐first‐order model (R^2^ = 0.9999), indicating dominant physical adsorption. In contrast, ACMEC and Honeywell fit the pseudo‐second‐order model, while DUPONT aligned with the pseudo‐first‐order model. Despite differences in their kinetic models, the initial adsorption rate (v_0_) of PVDF‐HFP exceeded that of activated carbons by 7,000–23,000 times, demonstrating ultrafast adsorption. Additionally, its half‐life (t_1/2_ = 0.25 min) was 1,500–7100 times shorter than those of activated carbons (t_1/2_ = 381–1753 min), further confirming its rapid kinetics. We also tested the adsorption behavior of activated carbon under ultrasonic conditions (Figure S2, Supporting Information). Within 120 s of ultrasound treatment, the adsorption of RhB by Honeywell, DUPONT, and ACMEC activated carbon was ≈42.8%, 85.1%, and 10.9%, respectively, all of which were significantly lower than that of PVDF‐HFP. Moreover, PVDF‐HFP demonstrates stable adsorption behavior, without the sustained ultrasonic‐induced desorption observed in materials like DUPONT AC. We subsequently investigated the adsorption capacity of PVDF‐HFP materials compared to commercial activated carbon by calculating the adsorption capacity per unit area. Although PVDF‐HFP possesses a relatively low specific surface area of ≈4 m^2^ g^−1^, it exhibited an adsorption capacity of 208.6 mg m^−2^, significantly exceeding the typical range of 0–60 mg m^−2^ reported for activated carbon (Ref.[18], and our results in Figure 1e; Table S3, Supporting Information). This discrepancy may originate from the multilayer physisorption behavior of PVDF‐HFP, where pollutants accumulate dynamically through electrostatic interactions or intermolecular forces across accessible surface sites, differing from the saturation‐limited monolayer adsorption observed in activated carbon.^[^ ^19^ ^]^

Dynamic adsorption experiments were conducted to verify the applicability of PVDF‐based adsorbents in practical water treatment. The prepared materials (1.45 g, cut into slices) were placed in a U‐shaped tube (Figure S3, Supporting Information), and a peristaltic pump (28 mL min^−1^) was used to introduce 300 mL of RhB solution into the system. Live video documentation captured the entire adsorption process (Movie S1, Supporting Information), showing that the colored RhB solution flowing through PVDF‐HFP in the U‐shaped tube was instantly transformed into a colorless liquid (Figure 1f). To quantify the adsorption process, we tested the cycling adsorption performance of a 300 mL RhB solution, achieving nearly complete adsorpion within 30 min (Figure S4, Supporting Information). In contrast to that solution pH significantly affects pollutant adsorption in other reports, there were no significant differences in the adsorption rate of RhB within the initial pH range of 3–9 (Figure 1g). Meanwhile, the RhB adsorption was not influenced by the natural water bodies, including tap water, Siyuan Lake, and Hanze Lake, demonstrating that matters in complex environments do not hinder rapid adsorption (Figure 1h). Moreover, the adsorption performance of PVDF‐HFP was systematically evaluated under varying ultrasonic frequencies, revealing a significantly enhanced efficiency under low‐frequency ultrasound (Figure S5, Supporting Information).

We further tested the versatility of the PVDF‐HFP material in absorbing other molecules. The results showed that over 95% adsorption efficiency was achieved for crystal violet (CV) and methylene blue (MB), and ≈80% efficiency for methyl orange (MO) within 5 min of ultrasonic treatment (Figure 1i). Furthermore, the adsorption performance of PVDF‐HFP materials toward key pollutants was evaluated. Levofloxacin (LEV), as a commonly used antibiotic, is a significant environmental pollutant that threatens aquatic ecosystems.^[^ ^20^ ^]^ Removing LEV is notoriously challenging due to its high solubility and chemical stability. Current removal methods, such as photocatalysis and adsorption columns, are ineffective due to slow removal rates, typically requiring over 30 min (Table S4, Supporting Information). Contrarily, Figure 1j presents the UV–vis spectra of the LEV adsorption process, demonstrating a rapid decrease of over 94% within 5 min. Meanwhile, PFOA (perfluorooctanoic acid) is resistant to degradation due to its strong carbon─fluorine bonds and chemical stability, leading to environmental accumulation. It poses significant health risks, including liver damage, immune system suppression, and an increased risk of certain cancers.^[^ ^21^ ^]^ Our tests for PFOA (in 5 mL solution) by PVDF‐HFP (0.046g) demonstrated a rapid reduction in concentration, with levels dropping by ≈90% within 5 min and declining from 5500 to ≈120 ppb within 20 min (Figure 1k). It should be mentioned that reported strategies often require hours or even days, leaving residual concentrations in the ppm range (Table S5, Supporting Information).^[^ ^22^ ^]^

### 2.2 Desorption and Regeneration of PVDF‐HFP

**Figure 2:**
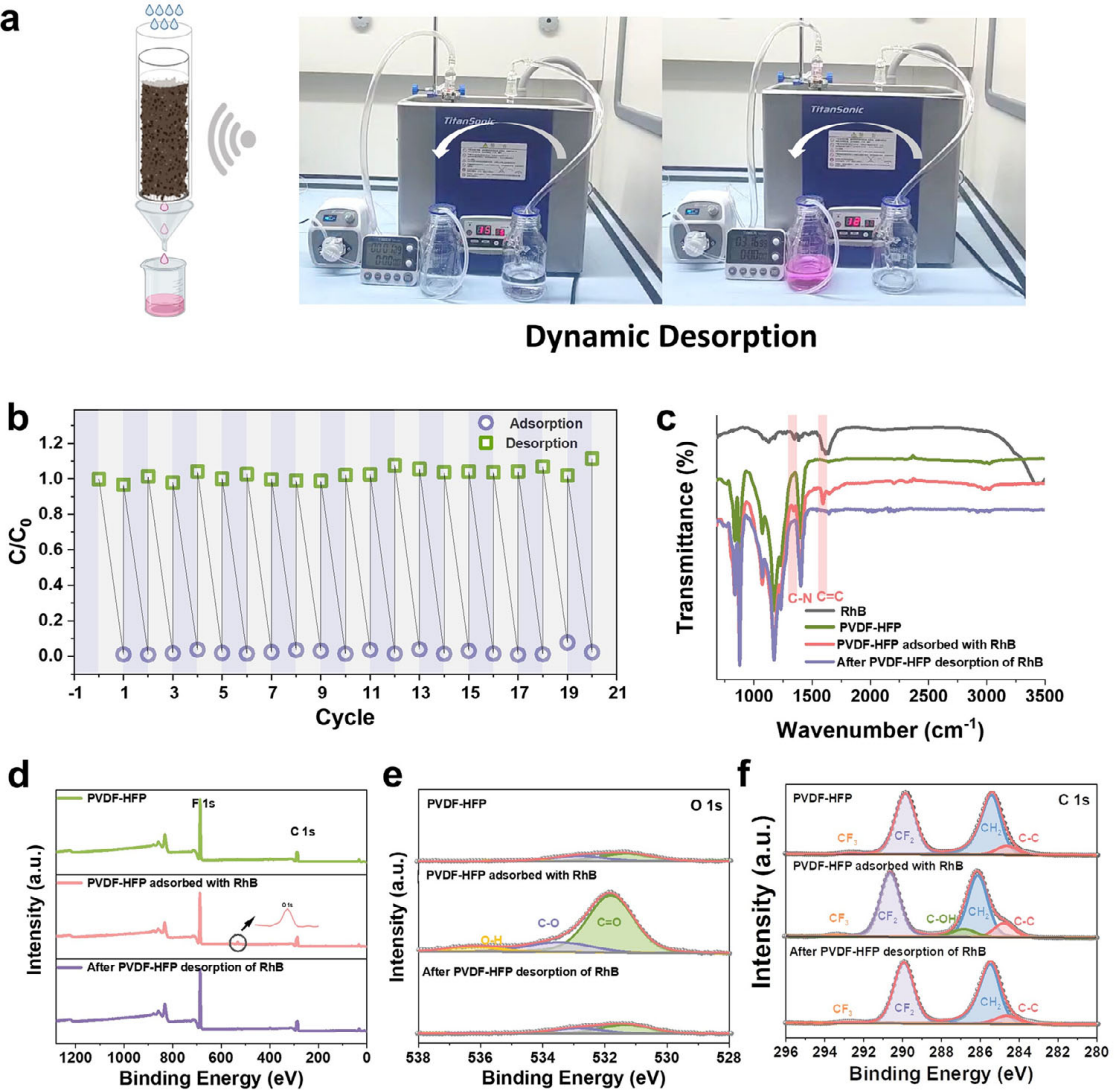
Adsorption–Desorption Cyclic Regenerability of the Material. a) Dynamic desorption performance of PVDF‐HFP membranes. b) The RhB removal efficiency by PVDF‐HFP material after consecutive regeneration cycles. c) FTIR, d) XPS, and high‐resolution, e) XPS‐O 1s and f) XPS‐C 1s spectra of PVDF‐HFP membranes: before adsorption, after adsorption, and after desorption, respectively.

The ability to regenerate adsorbent materials is crucial for their practical applications. We employed the dynamic experiment to validate the desorption process (**Figure** **2**a). An acidic ethanol solution (0.02 mm HCl in ethanol) was prepared as the desorption solution under ultrasonic treatment. As shown in Movie S2 (Supporting Information), the acid ethanol efficiently extracted all RhB from the material into the eluent in 4 min. Adsorption–desorption cycling experiments were also conducted. The results demonstrated stable performance with no decline observed in at least 20 cycles (Figure 2b). To confirm the effective adsorption and desorption of RhB, we conducted FTIR spectral analysis of the PVDF‐HFP materials before and after adsorption, as well as after desorption (Figure 2c). The results showed the characteristic peaks at 1343 and 1596cm^−1^, corresponding to the C─N and C═C bonds of the RhB molecules, appeared after adsorption. Additionally, the spectra of the regenerated materials after desorption showed no significant differences compared to the pristine PVDF‐HFP materials, confirming the complete desorption.

Further characterization was performed using X‐ray photoelectron spectroscopy (XPS). A minor peak corresponding to the O element was detected in the RhB‐adsorbed PVDF‐HFP, confirming the successful adsorption of RhB (Figure 2d,e). The O peak increased after RhB adsorption and sharply decreased after desorption, confirming successful adsorption and removal of oxygen‐containing organics. In the high‐resolution XPS C1s spectrum (Figure 2f), RhB‐adsorbed PVDF‐HFP showed a new C‐OH peak and increased C‐C intensity. Additionally, a pronounced leftward shift of the CF₂ peak was observed in the adsorbent containing RhB, which returned to its original position after desorption. The shift of the XPS peak to higher binding energy implied the strengthening of chemical bonds or the occurrence of electron transfer.

### 2.3 Ferroelectricity is Necessary for the Adsorption

**Figure 3:**
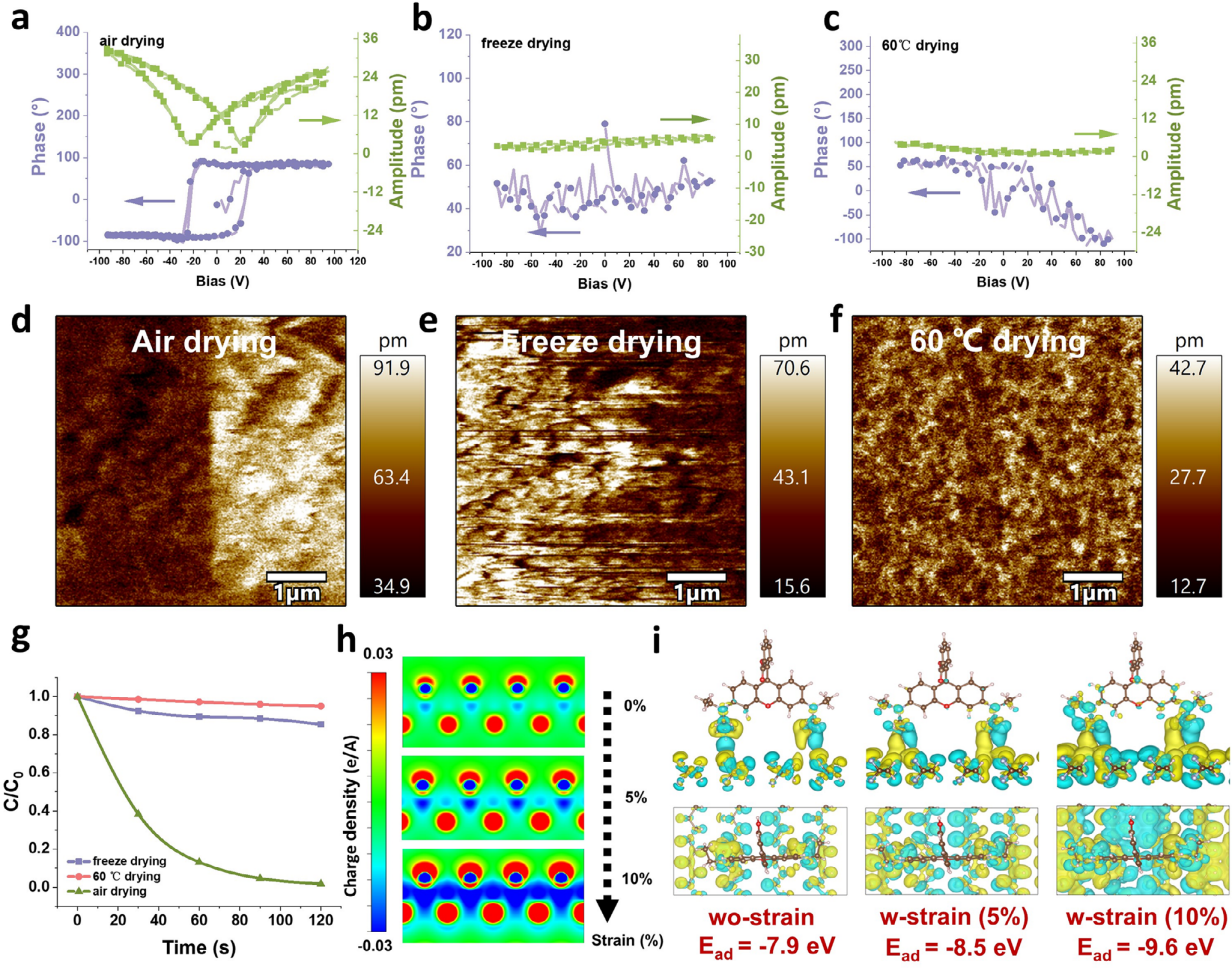
Effect of piezoelectricity on adsorption performance. a–c) The corresponding PFM phase hysteresis and amplitude butterfly loops, d–f) Out‐of‐plane PFM amplitude images for the assembled PVDF‐HFP material measured after applying ±60 V voltage, and g) Adsorption efficiency of PVDF‐HFP material with different drying methods. h) Adsorption energy of PVDF molecule and RhB under different stress states. i) 2D charge density diagram of PVDF with increasing stress.

Our results indicated that the adsorption–desorption of PVDF‐HFP undergoes a distinct mechanism. In this work, we employed wet PVDF‐HFP films stored in water/ethanol solutions, which is different from other studies utilizing high temperatures to dry PVDF‐based materials.^[^ ^23^ ^]^ The drying methods may affect the ferroelectric properties of the polymers.^[^ ^24^ ^]^ Therefore, we prepared PVDF‐HFP using freeze‐drying, drying at 60 °C, and air‐drying methods. The ferroelectric properties and adsorption capacities were characterized. PFM testing demonstrated that air‐dried PVDF‐HFP materials exhibited the characteristic phase hysteresis loops and amplitude butterfly curves associated with ferroelectricity. In contrast, PVDF‐HFP materials dried using the other two methods did not show ferroelectric signals (**Figure** **3**a–c). We further conducted local switching tests by applying a bias voltage with a conductive PFM tip. Figure 3d–f and Figure S6 (Supporting Information) display the PFM amplitude and phase images of PVDF‐HFP materials after inscribing symmetric square patterns with reversed DC bias. The significant phase reversal observed in air‐dried materials confirms successful polarization switching, while freeze‐dried and oven‐dried materials showed no notable reversals. DSC and FTIR analysis confirmed that air‐dried PVDF‐HFP materials exhibit the highest crystallinity and β‐phase content, possibly contributing to the ferroelectricity (Figure S7a–c, Supporting Information). This enhanced β‐phase content in air‐dried samples is attributed to slow solvent evaporation at ambient temperature, which promotes conformational ordering into β‐phase‐specific TTT sequences. Despite comparable overall crystallinity across all drying methods, rapid solvent removal in the oven (60 °C) and freeze‐drying restricts molecular rearrangement, resulting in kinetically trapped phases with lower β‐phase purity.^[^ ^25^ ^]^ Next, the adsorption properties of the three types of PVDF‐HFP were assessed (Figure 3g). Remarkably, only the air‐dried one exhibited exceptional adsorption performance, which aligns with the ferroelectric properties. To elucidate the role of ferroelectricity, PVDF‐HFP films with varying β‐phase contents were prepared by adjusting drying temperatures. Although the β‐phase is commonly associated with ferroelectric behavior, no direct correlation with adsorption efficiency was observed (Figure S7, Supporting Information), indicating that β‐phase content alone is insufficient. In contrast, only samples exhibiting a clear ferroelectric response showed enhanced adsorption, underscoring ferroelectricity as a necessary condition for high adsorption efficiency.

Although ferroelectric materials can be polarized under ultrasound,^[^ ^26^ ^]^ our results raise the question of how the polarization assists the adsorption. We employed density functional theory (DFT) calculations. The β‐phase of PVDF‐HFP was selected as the model for our calculations to optimize computational efficiency, as the characterization results confirmed that the ferroelectric β‐phase is predominant. We evaluated the adsorption energies of RhB molecules without mechanical stimulation at various sites and identified the (100) crystal plane as optimal (Figure S8, Supporting Information). Then, a 0%–10% strain was applied to PVDF to simulate changes in charge density under ultrasonic stimulation (Figure 3h). The spontaneous polarization of PVDF along the b‐axis increases as strain increases, leading to a pronounced charge gradient.

Subsequently, the interaction between the PVDF‐HFP surface and RhB molecules under different stress conditions was calculated (Figure 3i). Under stress, the increased spontaneous polarization along the b‐axis resulted in a more significant surface charge density difference. PVDF also exhibits shorter bond lengths and closer interaction distances with RhB molecules under stress. Ultimately, the increased spontaneous polarization and reduced interaction distances decreased adsorption energy from −7.9 to −9.6 eV on the (001) plane, indicating the enhanced absorption possibility.

### 2.4 Ferroelectricity is Insufficient, and Surface Tension might be the Hinge

**Figure 4:**
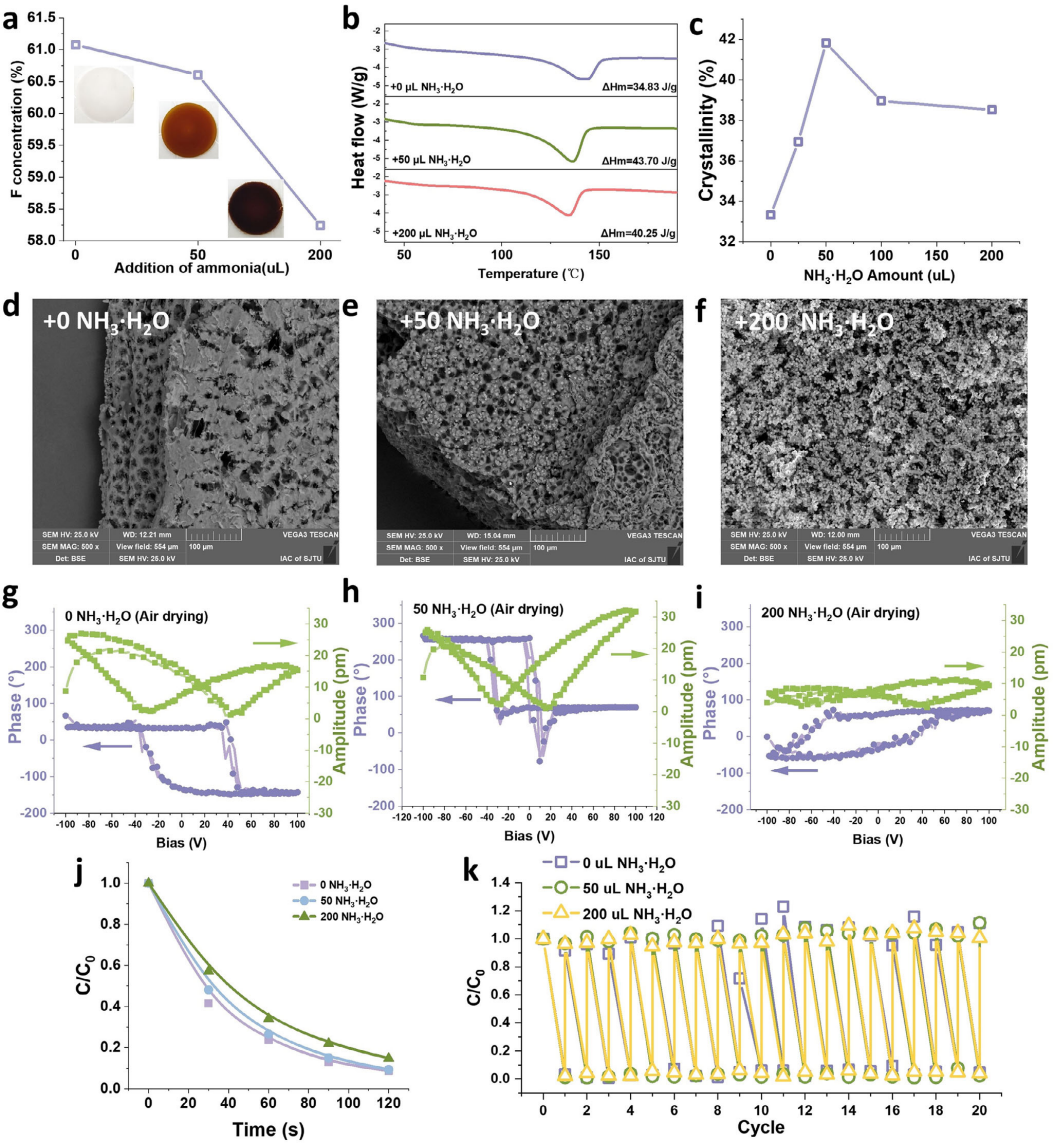
Effect of fluorine content on adsorption performance. a) Ion chromatography of F content, b) DSC curves, c) crystallization, d–f) SEM images, g)‐i) the corresponding PFM phase hysteresis and amplitude butterfly loops, j–k) and the adsorption performance of the PVDF‐HFP material with different ammonia concentrations.

To further evaluate whether there are any other factors, we adjusted the ammonia concentration in the preparation^[^ ^27^ ^]^ to assess its impact on PVDF‐HFP. As shown in **Figure** **4**a, ion chromatography confirmed that higher ammonia concentrations led to reduced fluorine levels in PVDF‐HFP. Meanwhile, we evaluated the influence of adding ammonia on the crystallinity of the PVDF membrane using differential scanning calorimetry (DSC) measurements, as shown in Figure 4b,c. The analysis indicated that crystallinity varied with ammonia concentration, reaching a peak at 50 µL before declining. In addition to defluorination and crystallinity, ammonia promotes local phase separation, initiating the formation of PVDF clusters in solution. These clusters act as nucleation sites and gradually grow into spherical particles during phase transitions.^[^ ^27a^ ^]^ As ammonia concentration increases, the morphology of PVDF‐HFP evolves from a porous structure to stacked spherical particles (Figure 4d–f). Besides, adding ammonia significantly influences the ferroelectric properties of the PVDF‐HFP materials. We employed a piezoelectric force microscope (PFM) to evaluate the ferroelectric properties of the PVDF‐HFP prepared with various ammonium hydroxide. All three samples exhibited phase hysteresis loops and amplitude butterfly curves. The piezoelectric coefficient of PVDF‐HFP varied with the amount of ammonia added, exhibiting d*_eff_* values ranging from 0.075 to 2.613 pm V^−1^ (Figure 4g–i). Unexpectedly, the variations caused by adding ammonia did not lead to a significant change in adsorption rate or recyclability (Figure 4j,k).

**Figure 5:**
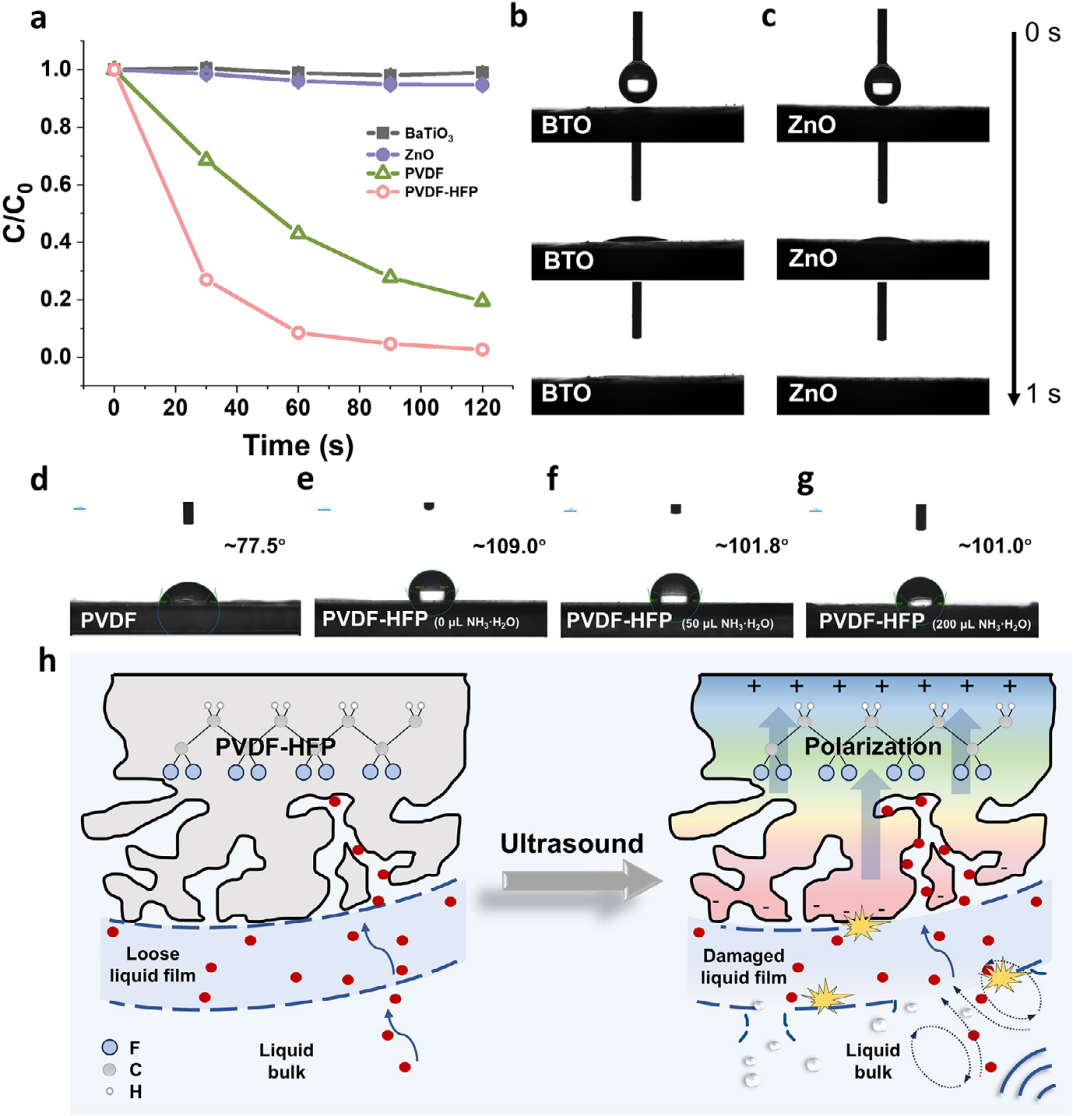
Effect of surface energy on adsorption mechanism. a) Adsorption performance of RhB by BTO and ZnO under ultrasound. The water contact angle of b) BTO and c) ZnO within 1 s. d–g) The water contact angle of PVDF and PVDF‐HFP with different ammonia concentrations. h) Diagram of the influence of surface energy on liquid film diffusion under ultrasound.

These results indicated that fluorine content, crystallinity, and microstructure did not affect adsorption. More unexpectedly, the ferroelectric polarization and butterfly curves are uncorrelated with the unchanged adsorption performance. This result indicates that the ferroelectric polarization did not affect the absorption linearly, and only the ferroelectricity might be insufficient. Therefore, we next selected barium titanate (BTO) and ZnO, the well‐known inorganic ferroelectric and piezoelectric materials, respectively, for the adsorption testing. However, neither BTO nor ZnO exhibited notable adsorption activity under identical experimental conditions (**Figure** **5**a). Meanwhile, ferroelectric PVDF (Figure S9, Supporting Information) achieved 80% RhB adsorption in 120s but remained less efficient than PVDF‐HFP (Figure 5a). This result further proved that ferroelectricity is not the only factor for ultrafast and efficient adsorption.

Considering that crossing the liquid film is essential for the adsorption,^[^ ^28^ ^]^ we hypothesize that surface energy might contribute to the ultrafast adsorption under ultrasound. The cavitation could alleviate the hindrance of liquid films to adsorbate diffusion and expose more active adsorption sites. Therefore, we calculated the diffusion coefficients of PVDF‐HFP. The results (Table S6, Supporting Information) revealed that ultrasonic assistance increased the liquid film diffusion coefficient by 207‐fold and the intraparticle diffusion coefficient by six‐fold. Under ultrasound, hydrophobic surfaces should have a higher possibility of forming cavitation than hydrophilic ones.^[^ ^29^ ^]^ Hence, we measured the water contact angle of the materials. BTO and ZnO show apparent affinity toward water droplets with a contact angle of 0° within 1 s (Figure 5b,c). This result indicates that their surface energy is equal to or greater than the surface tension of water (≥ 72.8 mN m^−1^). In contrast, the air‐dried PVDF displayed a contact angle of ≈77.5° (Figure 5d). Meanwhile, the air‐dried PVDF‐FHP sample without ammonia exhibited a contact angle of ≈109.0°. It slightly decreased to ≈101.8° and ≈101.0° for the samples with 50 and 200 µL of ammonia, respectively (Figure 5e–g). Interestingly, the contact angles of BTO, ZnO, PVDF, and PVDF‐HFP correlate well with their absorption ability, supporting the hypothesis that the hydrophobicity of PVDF‐based materials might be a crucial contributor.

Our results showed that lacking piezoelectricity or surface energy modulation resulted in an unsatisfactory outcome. Therefore, we proposed the mechanism of absorption enhancement (Figure 5h) ultrasonic stimulation disrupts the more vulnerable liquid film on low‐surface‐energy PVDF surfaces, facilitating the diffusion of solute molecules through the film onto the surfaces. Meanwhile, ultrasound triggers the generation of an intrinsic electric field in piezoelectric PVDF‐based materials, enabling direct and controlled solute absorption. Thus, the combined effects of ultrasound, ferroelectricity, and low surface energy synergistically promote rapid adsorption.

## 3 Conclusion and Outlook

This work discovered that PVDF‐HFP exhibited ultrafast and efficient adsorption–desorption behavior when exposed to ultrasound. The strategy is applicable to absorb various chemicals, including dyes (RhB, MB, MO, CV), antibiotics (LEV), and persistent contaminants (PFOA). We showed that ferroelectric materials are needed for the adsorption, but only ferroelectricity is insufficient. A hydrophobic surface might be another prerequisite. Meanwhile, the internal electric field generated under external force accelerates the adsorption process. These results suggested an alternative adsorption mechanism involving the disruption of the surface liquid film on low‐surface‐energy PVDF‐HFP to facilitate the diffusion and attachment of solute molecules.

Our tests for adsorbing Pb ions, tetracycline, and bacteria were unsuccessful. Based on current experiments, we are unable to conclusively determine which types of ions or molecules can be effectively adsorbed, likely due to the influence of multiple interrelated factors. First, hydrophobic interactions likely play a key role in driving adsorption onto the low‐surface‐energy PVDF‐HFP matrix. This is supported by the observed trend between adsorption efficiency and compound hydrophobicity (Figure S10, Supporting Information), as indicated by their n‐octanol/water partition coefficients (log P o/w):^[^ ^30^ ^]^ PFOA ≈ 6.3 > RhB ≈ 1.9 > CV ≈ 1.17 > MB ≈ −0.1 > LEV ≈ −0.39 > MO ≈ −0.66 > TC ≈ −1.19. The strong hydrophilicity of tetracycline (TC), attributed to its multiple polar functional groups (e.g., hydroxyl and amine), likely diminishes its ability to interact with the hydrophobic polymer surface. Second, the ultrasound‐assisted process may induce a polarized and negatively charged surface on the PVDF‐HFP material, which facilitates electrostatic attraction with strongly charged species. In contrast, weakly charged or zwitterionic molecules such as TC may exhibit limited electrostatic interactions, particularly under neutral pH conditions. Finally, the bulky and rigid tetracyclic structure of TC (≈1.2 nm) may impose steric hindrance to adsorption. In comparison, smaller or more planar molecules, such as LEV (≈0.8 nm) and RhB, with their relatively flat aromatic frameworks, may more readily access adsorption sites. Collectively, these observations indicate that adsorption behavior is governed by a complex interplay of factors, including molecular charge, hydrophobicity, polarity, and size.

Nonetheless, our strategy might have broad practical potential in multiple fields. Given that PVDF‐based ultrafilters have been widely used in water treatment, one possibility is to partly exert the functions of nanofiltration or reverse osmosis, simplifying the whole treatment chain. Our work also offers a compelling alternative to activated carbon adsorption, advanced oxidation processes (AOPs), and reverse osmosis, delivering a more efficient and sustainable solution for pollutant removal. In fine chemical production, impurities are often eliminated through adsorption, membrane separation, or chemical reactions, while drug purification in pharmaceuticals typically involves chromatography, crystallization, and membrane filtration.^[^ ^31^ ^]^ PVDF‐HFP, when combined with ultrasound, may significantly elevate the production efficiency.

## 4 Experimental Section

### Materials and Instruments

Poly(vinylidene fluoride‐co‐hexafluoropropylene) (Melt index: 9–15g/10min; Tm:132–136 °C, pellets), 1‐Methyl‐2‐pyrrolidinone (NMP, for HPLC, ≥99.5%) were purchased from Meryer Biochemical Technology Co. Ltd. Ammonia was purchased from Aladdin. Rhodamine B (AR) was purchased from Mackin. Activated carbons were purchased from ACMEC, DOPONT, and Honeywell.

The morphology of PVDF‐based adsorbents was obtained by scanning electron microscope (SEM, TESCAN, VEGA 3‐XMU (LaB6)). The crystal phase of the PVDF‐based materials was determined by powder X‐ray diffractometry (XRD, Aeris, Malvern Panalytical). UV–vis absorbance spectra were measured by a UV–vis spectrometer (AXIS UltraDLD, Shimazu). The X‐ray photoelectron spectra (XPS) were recorded by X‐ray Photoelectron Spectrometer (Nexsa, Thermo Fisher Scientific). The piezoelectric and ferroelectric properties of nanorods were carried out by an atomic force microscope (AFM, MFP‐3D, Asylum Research). Fourier Transform infrared spectroscopy (FT–IR) spectra were measured by a Fourier transform infrared spectrometer (Nicolet 6700, Thermo‐fisher). The fluorine content was measured using ion chromatography (Thermal Scientific ICS‐90) following combustion analysis. The crystallinity of PVDF was determined using differential scanning calorimetry (DSC, DSC2500).

### Fabrication of the PVDF‐Based Adsorbents

The preparation of PVDF‐based piezoelectric adsorbents is based on the humidity‐induced phase inversion method. 0.125 g PVDF‐based materials (PVDF, PVDF‐HFP, and PVDF‐TrFE) were dissolved in 1.188 mL N‐methyl‐2‐pyrrolidone (NMP) with heating, followed by the addition of 0–200 µL ammonia as an inert solvent to induce localized phase separation. The solution was then cast into molds and allowed to settle, producing films, microspheres, or fibers depending on the mold shape. The cast solution was then placed in a high‐humidity environment (>80% relative humidity) to induce phase separation and structural solidification.

### Adsorption of Pollutants by PVDF‐Based Adsorbents

One piece of PVDF‐based film was placed in a beaker, followed by the addition of a 100 mg L^−1^ pollutant solution. The beaker containing the adsorbent and pollutants was then subjected to ultrasonic irradiation (180 W, 35 kHz) without magnetic stirring. During the ultrasonic treatment, aliquots were sampled at 30‐s intervals and analyzed at the maximum absorption wavelength using a UV–vis spectrophotometer. The quantification of PFOA was conducted using an ultra‐performance liquid chromatography‐mass spectrometry (UPLC‐MS) system (Shimadzu Qp 2020 Nx Japan). Chromatographic separation was carried out on a C18 column maintained at 35 °C with a flow rate of 150 µL min^−1^. The mobile phase consisted of eluent A (2.5 mmol L^−1^ ammonium acetate in water) and eluent B (acetonitrile). The gradient program for eluent B started at 30%, increased to 70% over 4 min, returned to the initial 30% at 7 min, and was held at 30% for an additional 3 min to ensure system stability. Sample analysis was performed in negative ion mode using multiple reaction monitoring.

### Crystallization of PVDF‐Based Adsorbents

The following equation was employed to determine the crystallinity of PVDF prepared with different drying methods^[^ ^32^ ^]^:

(1) $$X_{c}=\left(\frac{\Delta H_{m}}{\Delta H_{m}^{\emptyset }}\right)\times 100\%$$

where Δ*H_m_* refers to the melting enthalpy of the material calculated using the DSC software, whereas $\Delta H_{m}^{\emptyset }$ indicates the melting enthalpy of 100% crystalline PVDF, which is 104.5 J g^−1^.

### DFT Computations

First principles energetic and electronic structure calculations were carried out within the framework of the density‐functional theory (DFT) using projector augmented wave (PAW) pseudopotentials as implemented in the Vienna ab initio simulation package (VASP). The generalized gradient approximation (GGA) formulated by Perdew, Burke, and Ernzerhof (PBE) was used as the exchange‐correlation functional. A kinetic energy cutoff of 400 eV for the plane wave basis set was generated. The Brillouin zone was sampled with Γ‐centered 1×1×1 k‐points. The energy and force convergence criteria were set to 10‐5 eV and 0.01 eV Å^−1^. Compression stress was applied to the b‐axis to simulate the piezoelectric phase.

### Adsorption Kinetics Model Analysis

The pseudo‐first‐order (PFO) kinetic model is as follows:

(2) $$q_{t}=q_{e}\left(1-e^{-k_{1}t}\right)$$

(3) $$\ln\left(q_{e}-q_{t}\right)=lnq_{e}-k_{1}t$$

(4) $$v_{0}=k_{1}q_{e}$$

(5) $$t_{1/2}=\frac{k_{1}}{ln2}$$

The pseudo‐second‐order (PSO) kinetic model is as follows:

(6) $$q_{t}=\frac{k_{2}q_{e}^{2}t}{1+k_{2}q_{e}t}$$

(7) $$\frac{t}{q_{t}}=\frac{1}{k_{2}q_{e}^{2}}+\frac{t}{q_{e}}$$

(8) $$v_{0}=k_{2}q_{e}^{2}$$

(9) $$t_{1/2}=\frac{1}{k_{2}q_{e}}$$

Here, *k*_1_ and *k*_2_ represent the rate constants of the pseudo‐first‐order (PFO) and pseudo‐second‐order (PSO) adsorption kinetic models, respectively. *q_e_* (mg g^−1^) denoted the adsorption capacity at equilibrium, while *q_t_* (mg g^−1^) was the amount of adsorbate adsorbed at any given time (in minutes). *v*_0_ represents the initial sorption rate, and *t*_1/2_ refers to the adsorption half‐life.^[^ ^33^ ^]^

### Diffusion Rate Constant Calculation

To investigate whether intraparticle diffusion was the rate‐limiting step of the adsorption process, the Weber–Morris equation was applied:^[^ ^34^ ^]^

(10) $$q_{t}=k_{id}t^{0.5}+C$$

Here, *q_t_* (mg·g^−1^) represents the amount of adsorbate adsorbed onto the adsorbent at time t; k_id_ is the intraparticle diffusion rate constant (mg·g^−1^·min^−0.5^), and C is a constant.

The liquid film diffusion rate constant was calculated by the following equation:

(11) $$\ln\left(1-\frac{q_{t}}{q_{e}}\right)=-k_{F}t$$

Here, *q_e_* represents the adsorption capacity at equilibrium, and *k_F_* is the liquid film diffusion rate constant.

## Conflict of Interest

The authors declare no conflict of interest.

## Supporting information



Supporting Information

Supplemental Movie 1

Supplemental Movie 2

## Data Availability

The data that support the findings of this study are available from the corresponding author upon reasonable request.
