# Supplementary material for: Ultrasound‐Assisted for Super‐Rapid and High‐Efficient Adsorption and Desorption
Source: Adv Sci (Weinh). 2025 May 28;12(31):e04905. doi: 10.1002/advs.202504905 (PMC12376586; doi:10.1002/advs.202504905)
Supplement: Supplementary file 1 — Supporting Information [file ADVS-12-e04905-s003.docx]

Supporting Information

**Ultrasound-Assisted Super-Rapid and High-Efficient Adsorption and Desorption**

*Yijun Han, Quanjie Lv, Linxuan Zhang, Yuruo Zhang, Xinyue Yu, Yongjie Wu, Jing Chu, Gengxin Zhang, Kang Sun, Ke Tao**

**
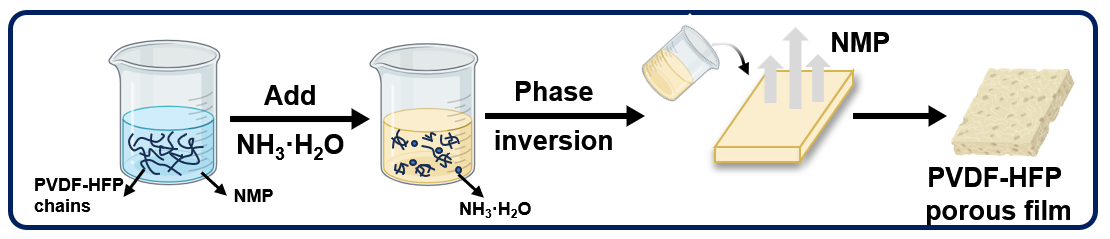
**

**Figure. S1.**

Preparation process of PVDF-based film.


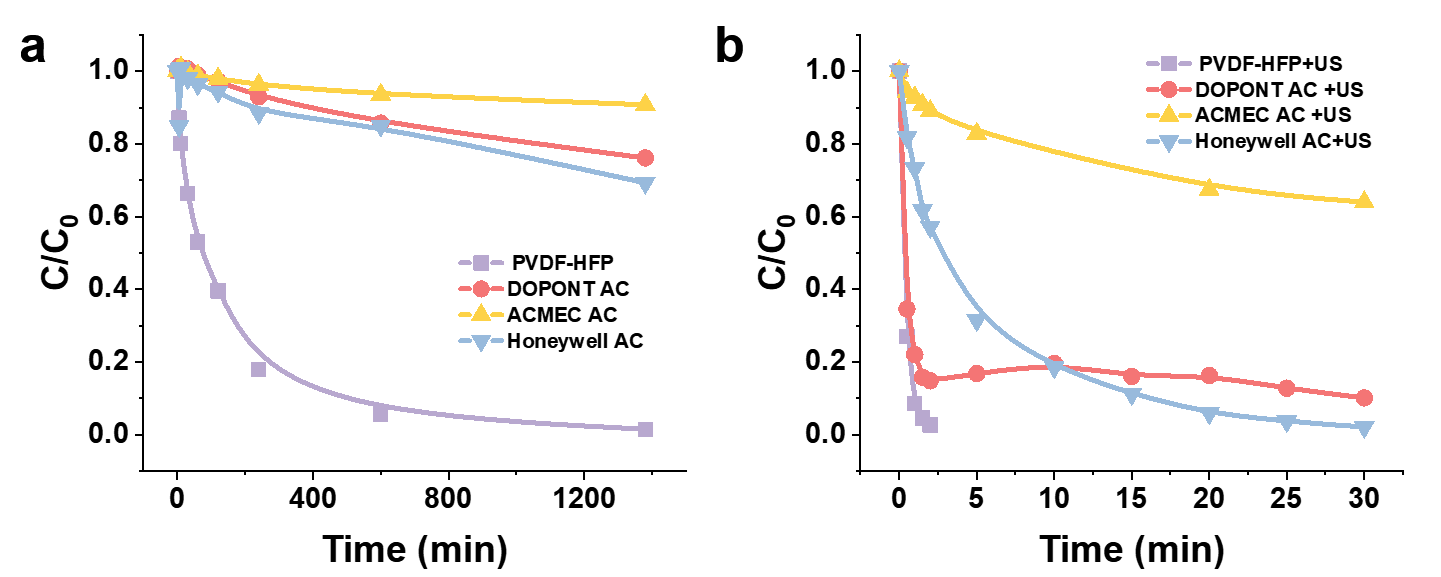


**Figure. S2.**

Time-dependent adsorption of RhB by PVDF-HFP film and activated carbon under a) static conditions and b) ultrasonic irradiation.


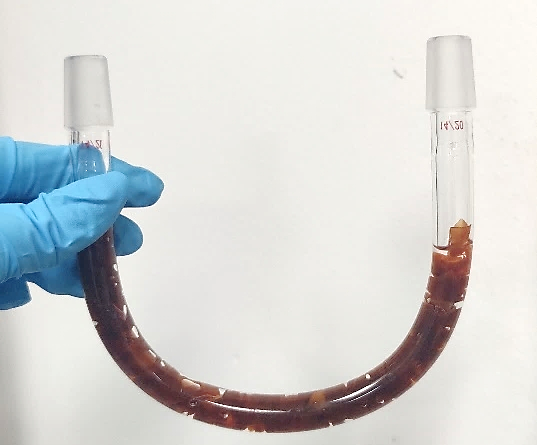


**Figure. S3.**

Picture of U-shaped tube in dynamic adsorption experiment.


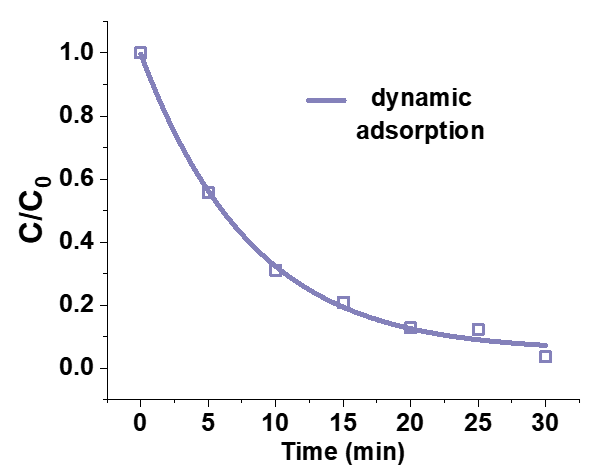


**Figure. S4.**

Time dependent dynamic adsorption curve.


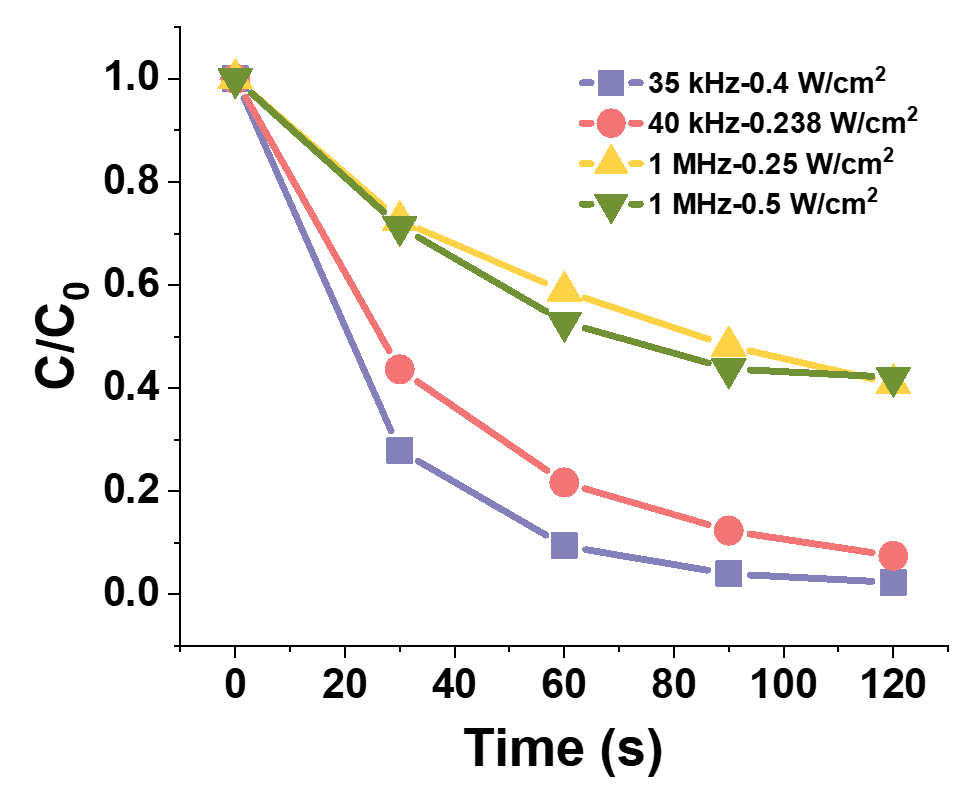


**Figure. S5.**

Time-dependent adsorption of RhB (100 ppm) by PVDF-HFP material at different ultrasonic frequencies.


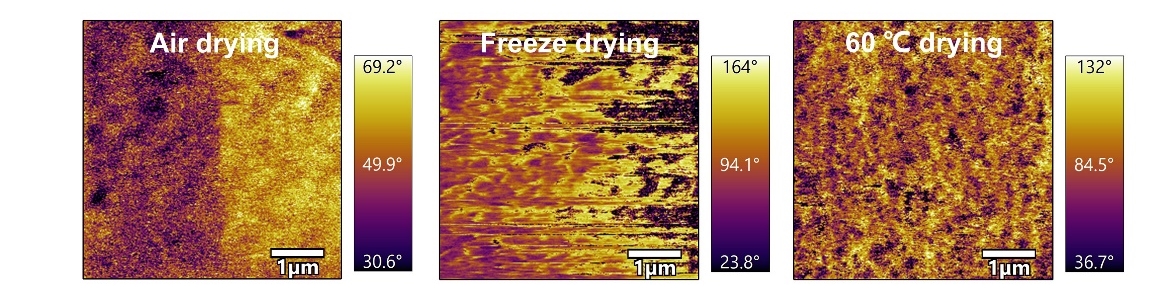


**Figure. S6.**

The out-of-plane PFM phase images for the assembled PVDF-HFP material measured after applying ±60 V voltage

**
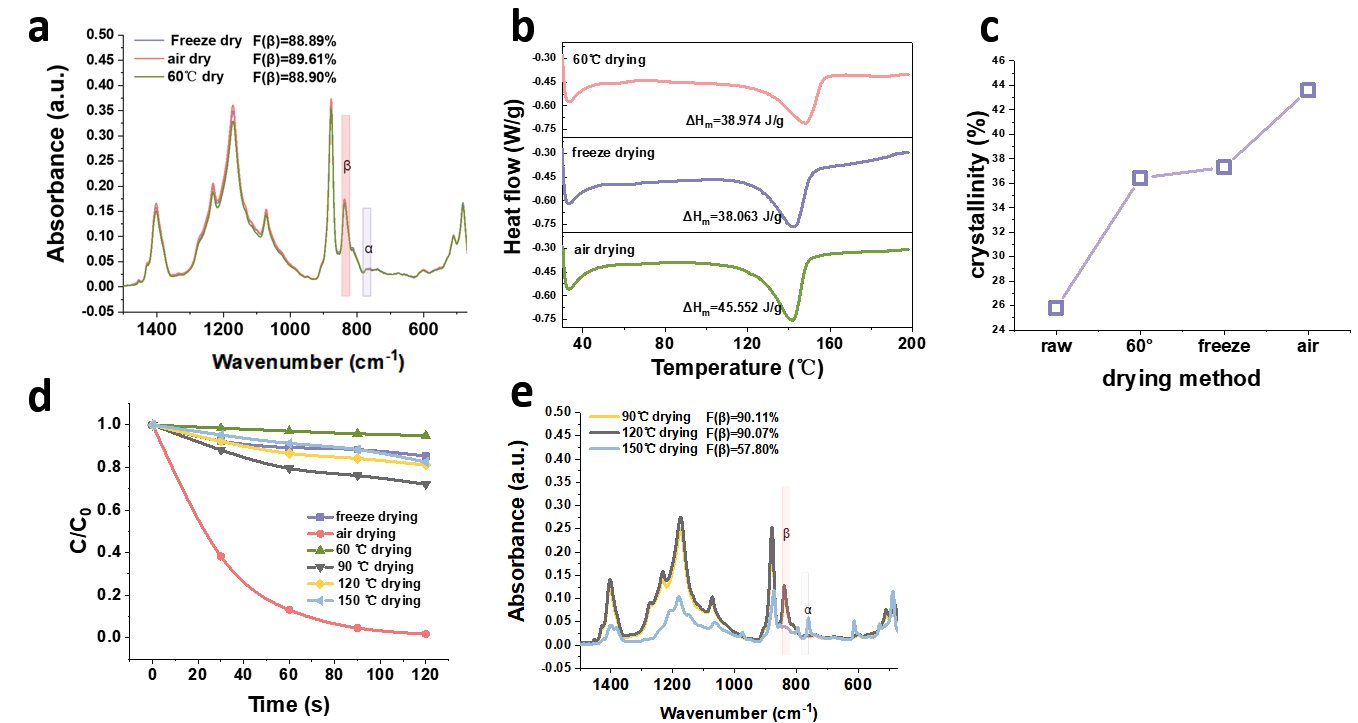
**

**Figure. S7.**

FTIR spectra, crystallinity and adsorption efficiency of PVDF-HFP film with different drying methods.


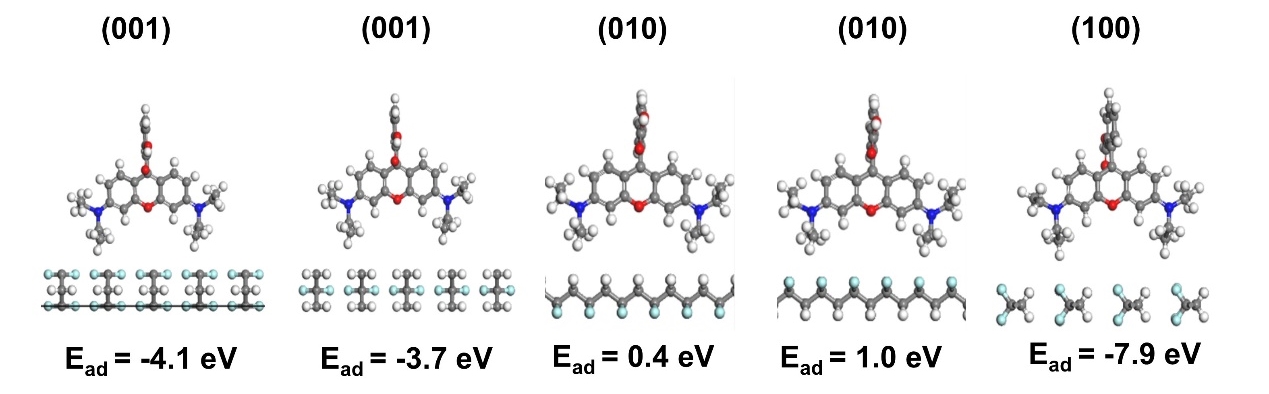


**Figure. S8.**

Adsorption energy calculation at different adsorption sites.


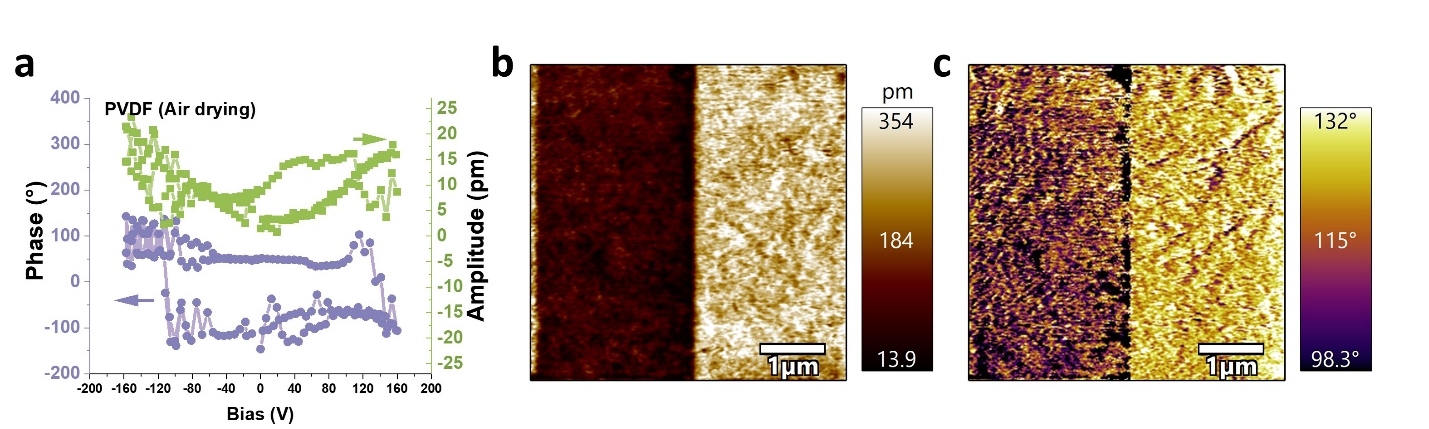


**Figure. S9.**

Ferroelectric properties of PVDF.


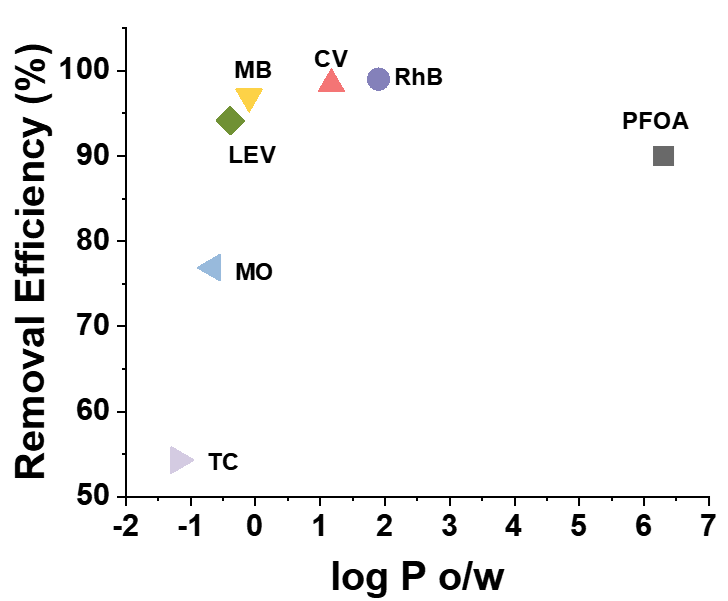


**Figure. S10.**

Removal efficiency of PVDF-HFP material under 5-minute ultrasonication for substances with varying logP values.

**Table S1.**

Technical parameters for RhB removal across various treatment techniques.

| Matericals | removal type | removal efficiency (%) | rate constant | Ref |
| --- | --- | --- | --- | --- |
| periodic mesoporous organosilicas | adsorption | 91.5% whthin 12 h (100 ppm) | - | ^[1]^ |
| activated carbon from white sugar | adsorption | 98.28% within 12 min (50 ppm) | k_1_=0.3919 min^-1^  k_2_=7.08×10^-3^ g·mg^-1^·min^-1^ | ^[2]^ |
| Artocarpus odoratissimus Leaves | adsorption | ~55% 3 h (100 ppm) | k_1_=0.02 min^-1^  k_2_=1.274 g/mol·min | ^[3]^ |
| MoS_2_ | adsorption | ~70% wihin 120 min (10 ppm) | k=0.01151 min^-1^ | ^[4]^ |
| Gelation/CuS/PVA | photocatalysis | 81% within 80 min (100 ppm) | - | ^[5]^ |
| BiOCl | photocatalysis | 35% within 6 min (20 ppm) | - | ^[6]^ |
| Glucose-BiOCl | Adsorption-photocatalysis | 100% within 6 min (20 ppm) | - | ^[6]^ |
| PVDF-HFP | Ultrasound-adsorpion | ~100% within 2 min (100 ppm) | k_1_ =2.775 min^-1^  k_2_= 0.3414 g·mg^-1^·min^-1^ | This work |

**Table S2.**

Adsorption kinetic parameters of pseudo-first-order and pseudo-second-order models for different adsorbents.

| Model | Parameters | PVDF-HFP+US | PVDF-HFP | ACMEC AC | Honeywell AC | DUPONT AC |
| --- | --- | --- | --- | --- | --- | --- |
| Pseudo-first-order (PFO) | k_1_ (min^-1^) | 2.7753 | 0.011 | 0.00182 | 0.00101 | 0.000897 |
|  | qe (mg/g) | 10.3985 | 9.6523 | 1.0313 | 4.1073 | 3.4812 |
|  | R^2^ | 0.9999 | 0.9679 | 0.9976 | 0.9675 | 0.9961 |
|  | v_0_ (mg·g^-1^·min^-1^) | 28.8590 | 0.1062 | 0.0019 | 0.0041 | 0.0031 |
|  | t_1/2_ | 0.2498 | 63.0134 | 380.8501 | 686.2843 | 772.7307 |
| Pseudo-second-order (PSO) | k_2_ (g·mg^-1^·min^-1^) | 0.3414 | 0.00142 | 0.00103 | 0.000126 | 0.000101 |
|  | qe (mg/g) | 11.8503 | 10.6166 | 1.427 | 6.0095 | 5.6255 |
|  | R^2^ | 0.9986 | 0.9887 | 0.9982 | 0.971 | 0.9956 |
|  | v_0_ | 47.9427 | 0.1601 | 0.0021 | 0.0046 | 0.0032 |
|  | t_1/2_ | 0.2472 | 66.3325 | 680.3600 | 1319.927 | 1753.25 |

**Table S3.**

Specific surface area, adsorption capacity, and adsorption capacity per unit surface area of different adsorbents.

| Adsorbents | Specific surface area (m^2^/g) | Adsorption capacity Q (mg/g) | Q/ Specific surface area (mg/m^2^) |
| --- | --- | --- | --- |
| Air-dried PVDF-HFP film | 4.083 | 851.6225 | 208.5776 |
| ACMEC AC | 53.747 | 769.5534 | 14.31807 |
| Honeywell AC | 209.217 | 3818.446 | 18.25113 |
| DUPONT AC | 31.423 | 1907.324 | 60.69835 |

**Table S4.**

Parameters of materials used for removal of LEV in various removal techniques.

| Matericals | removal type | removal efficiency (%) | rate constant | Ref |
| --- | --- | --- | --- | --- |
| Ag-AC | adsorption | - | k_1_=0.071 min^-1^  k_2_=7.07×10^-4^ g·mg^-1^·min^-1^ | ^[7]^ |
| Fe_3_O_4_ | adsorption | 80.1% within 240 min (2.5 ppm) | k_1_=1.12×10^-2^ min^-1^  k_2_=4.737×10^-2^ g·mg^-1^·min^-1^ | ^[8]^ |
| Biochar-derived [porous carbon nanosheets](https://www.sciencedirect.com/topics/materials-science/porous-carbon-nanosheets) | adsorption | 99.64% within 10 min (30 ppm) | k_1_=0.45 min^-1^  k_2_=5.67×10^-2^ g·mg^-1^·min^-1^ | ^[9]^ |
| CMNFs-MMT | adsorption | 89% within 60 min | k_1_=0.0576 min^-1^  k_2_=0.0019 g·mg^-1^·min^-1^ | ^[10]^ |
| Ag/AgBr/BiOBr | photocatalysis | 74% within 90 min (10 ppm) | k=0.01659 min^-1^ | ^[11]^ |
| ZnO/KNTs | photocatalysis | 99% within 30 min (10 ppm) | k=0.0237~0.152 min^-1^ | ^[12]^ |
| (BiOBr)_x_(Bi_7_O_9_I_3_)_1-x_ | photocatalysis | 95.4% in 120 min (50 ppm) | k=0.0286 min^-1^ | ^[13]^ |
| Au-Pd/TiO_2_ | photocatalysis | 95% within 60 min (5 ppm) | k=0.0159 min^-1^ | ^[14]^ |
| MoS_2_/TiO_2_ | photoelectrocatalysis | 100% within 180 min | k=2×10^-2^ min^-1^ | ^[15]^ |
| PMS assisted MoS_2_/C | sonophotocatalysis | 100% within 80 min (10 ppm) | k=0.0702 min^-1^ | ^[16]^ |
| PVDF-HFP | Ultrasound-adsorption | 94% within 5 min (5 ppm) | k_1_ =0.9425 min^-1^  k_2_= 1.8644 g·mg^-1^·min^-1^ | This work |

**Table S5.**

Technical parameters for PFOA removal across various treatment techniques.

| Matericals/Methods | removal type | Time | removal efficiency | rate constant | Ref |
| --- | --- | --- | --- | --- | --- |
| Granular activated carbon (GAC) | adsorption | 24 h | 50 ppm to 1 ppm | k_2_=0.032 g·mg^-1^h^-1^ | ^[17]^ |
| Anion-exchange resions (AERs) | adsorption | 2 h | 50 ppm to 0.5 ppm | k_2_=0.633 g·mg^-1^h^-1^ | ^[17]^ |
| TiO_2_ | photocatalysis | 7 h | 60 ppm to 41.3 ppm | 0.0581 h^-1^ | ^[18]^ |
| Ag-TiO_2_ | photocatalysis | 7 h | 60 ppm to 25.38 ppm | 0.1257 h^-1^ | ^[18]^ |
| Pd-TiO_2_ | photocatalysis | 7 h | 60 ppm to 3.48 ppm | 0.4369 h^-1^ | ^[18]^ |
| Pt-TiO_2_ | photocatalysis | 5 h | 60 ppm to undetectable (measured by UPLC-MS) | 0.7267 h^-1^ | ^[18]^ |
| TiO_2_+HClO_4_(0.075 M) | Photocatalysis | 7 h | 50 ppm to 7 ppm | 0.19 h^-1^ | ^[19]^ |
| Al-Zn electrodes (9 V+500 rpm) | Electrocoagulation | 60 min | 1000 ppb to 7 ppb | - | ^[20]^ |
| KI+UV | Photo-reduction | 14 h | 10.352 ppm to 0.114 ppm | k=0.438 h^-1^ | ^[21]^ |
| - | γ-irradiation | 6 h | 20 ppm to 0.02 ppm | k=0.67 h^-1^ | ^[22]^ |
| PVDF-HFP+ultrasound | Ultrasound-adsorption | 20 min | 5500 ppb to 120 ppb | k_1_=30.02 h^-1^  k_2_=0.164 g·mg^-1^h^-1^ | This work |

**Table S6.**

Intraparticle diffusion and liquid film diffusion model rate constant.

|  | Liquid film diffusion k_F_ （min^-1^） | R^2^ of k_F_ | intraparticle diffusion k_id_ g/(mg·min^0.5^) | R^2^ of k_id_ |
| --- | --- | --- | --- | --- |
| PVDF-HFP^#^ | 0.00661 | 0.97 | 0.564 | 0.991 |
| PVDF -HFP+US^*^ | 1.3675 | 0.954 | 3.63 | 0.868 |
| */# | 206.8835098 | - | 6.436170213 | - |

**Movie S1.**

Dynamic adsorption movie.

**Movie S2.**

Dynamic desorption movie.

[1] Y. Zhao, Y. Liu, L. Jiang, *New J. Chem.* **2024**, 48, 4109.

[2] W. Xiao, Z. N. Garba, S. Sun, I. Lawan, L. Wang, M. Lin, Z. Yuan, *Journal of Cleaner Production* **2020**, 253, 119989.

[3] N. A. H. Mohamad Zaidi, L. B. L. Lim, N. Priyantha, A. Usman, *Arabian Journal for Science and Engineering* **2018**, 43, 6011.

[4] R. Thayil, S. Cherukulappurath, *Nano Express* **2023**, 4.

[5] A. A. Al-Kahtani, *Journal of Biomaterials and Nanobiotechnology* **2017**, 08, 66.

[6] Z. Li, B. Ma, X. Zhang, Y. Sang, H. Liu, *Environ Res* **2020**, 182, 109077.

[7] Ş. Karadirek, H. Okkay, *J. Taiwan Inst. Chem. Eng.* **2019**, 105, 39.

[8] M. H. Al-Jabari, S. Sulaiman, S. Ali, R. Barakat, A. Mubarak, S. A. Khan, *J. Mol. Liq.* **2019**, 291, 111249.

[9] D. Yang, J. Li, L. Luo, R. Deng, Q. He, Y. Chen, *Chem. Eng. J.* **2020**, 387, 124103.

[10] J. Li, J. Tao, C. Ma, J. Yang, T. Gu, J. Liu, *RSC Adv.* **2020**, 10, 42038.

[11] G. Gupta, A. Kaur, A. S. K. Sinha, S. K. Kansal, *Mater. Res. Bull.* **2017**, 88, 148.

[12] M. R. Abukhadra, A. Helmy, M. F. Sharaf, M. A. El-Meligy, A. T. Ahmed Soliman, *J Environ Manage* **2020**, 271, 111019.

[13] S. G. Fard, M. Haghighi, M. Shabani, *Appl. Catal. B* **2019**, 248, 320.

[14] Q. Chen, Y. Xin, X. Zhu, *Electrochim. Acta* **2015**, 186, 34.

[15] L. Zeng, X. Li, S. Fan, Z. Yin, M. Zhang, J. Mu, M. Qin, T. Lian, M. Tadé, S. Liu, *Electrochim. Acta* **2019**, 295, 810.

[16] L. Zeng, S. Li, X. Li, J. Li, S. Fan, X. Chen, Z. Yin, M. Tadé, S. Liu, *Chem. Eng. J.* **2019**, 378, 122039.

[17] T. Obal, A. Robinson, C. E. Brown, K. Volchek, Y. Yao, *Water Sci. Technol.* **2014**, 70, 1983.

[18] M. Li, Z. Yu, Q. Liu, L. Sun, W. Huang, *Chem. Eng. J.* **2016**, 286, 232.

[19] S. C. Panchangam, A. Y.-C. Lin, K. L. Shaik, C.-F. Lin, *Chemosphere* **2009**, 77, 242.

[20] Y. Liu, X.-M. Hu, Y. Zhao, J. Wang, M.-X. Lu, F.-H. Peng, J. Bao, *Chemosphere* **2018**, 201, 303.

[21] Y. Qu, C. Zhang, F. Li, J. Chen, Q. Zhou, *Water Res.* **2010**, 44, 2939.

[22] Z. Zhang, J.-J. Chen, X.-J. Lyu, H. Yin, G.-P. Sheng, *Scientific Reports* **2014**, 4, 7418.
